# An Inquiry, Critical and Experimental, into the Pathology of Fever

**Published:** 1853-05

**Authors:** N. S. Davis

**Affiliations:** Professor of Pathology, Practice of Medicine, and Clinical Medicine, in Rush Medical College; and one of the Physicians of the Ill. Gen. Hospital


*An Inquiry, Critical and Experimental, into the Pathology of Fever.* By N. S Davis, M. D., Professor of Pathology, Practice of Medicine, and Clinical Medicine, in Rush Medical College; and one of the Physicians of the Ill. Gen. Hospital.

"A correct Physiology must form the basis of all sound Medical Philosophy."

Since the earliest dawnings of Medical Science, no class of diseases has attracted more attention, or their discussion occupied a wider space in the literature of our profession, than Fevers.

Occurring, in some form, in every climate, and often assuming the most serious degree of severity; their symptoms, causes and nature, have constituted subjects of the most careful study; and on them have been founded most if not all, the great systems or hypotheses that have swayed the minds of the profession, during the last two thousand years. With a knowledge of these facts, it may seem presumptuous to present for the consideration of the profession, another essay on a subject so long the study of the most enlightened minds that have adorned the pages of medical literature. And yet, when we examine with care, we shall find very few subjects connected with practical medicine involved in greater obscurity, or concerning which the knowledge to be gained from recent authors is less satisfactory. Even the elementary question, What is fever? remains *ad huc sub judice.* For while modern inquirers have entered into the most minute and extensive researches in regard to the causes, phenomena, differences, and post mortem appearances, presented in the different forms of fever, they have left the question, as to its essential nature, very nearly where it was a century since. Hence says Dr. Geo. B. Wood, "As to the real nature of the fever, (Typhoid) we are in the dark, as we are, in fact, in relation to all the essential fevers."* Again he says, "Theorists have failed in endeavoring to trace the complicated disorders of fever to some common source, and to point out a particular succession, a particular and necessary line of march, in the progress of the affection. The universal disturbance of function, which consti-

* See Wood's Practice, vol. i, page 337.

Reel; 78-10-5 CONT.

tutes the disease may be brought about in various ways; and the starting point may be entirely different in different cases. Yet, among the great majority of cases, there is a close analogy in the mode of onset, which must be ascribed to some common principle. Whether the fever is idiopathic or symptomatic, the first decided step towards its formation, seems to be some morbid impression upon the nervous system, and this impression seems to be of a depressing nature. The phenomena immediately preceding, and those attendant on the chill, are for the most part unequivocally those of depression. The whole nervous system appears to have received a shock from the cause, cramping and for a time deadening its energies. Along with the diminished exercise of the nervous function, is necessarily a diminution of all those functions dependent upon it. We may thus partially explain the condition of the chill; *but there is something more which we do not fathom;* something in which the chill of fever differs from other instances of nervous depression. Upon the principles which have already been explained, the general prostration is followed by reaction, and the fever is then established. But there is here also something more than mere reaction.—There is the continued action of the cause, a diversified play of sympathies in one case, a widely pervading influence from some unknown agent in another; and fever is not purely, as some have maintained, the *resilience* of the bowed down system. *To unravel this complicated web, is, in the present state of our knowledge, impossible.*"*

Dr. Holland likewise observes: "We can scarcely touch on this subject of fevers, especially the idiopathic, without finding in it a bond by which to associate together numerous forms of disease; but, withal, *a knot so intricate that no research has hitherto succeeded in unraveling it.*"

With the exception of Clutterbuck, Broussais, and their disciples, who regard all fevers as dependent on local inflammation, I might quote similar acknowledgements from almost every writer for the last half century. The advocates of the inflammatory origin of fevers are now comparatively very few. Equally small is the number of those who adhere to the systems of Brown, Darwin, Cullen or

* See Wood's practice, vol. i, page 109.

Rush. It would seem that modern investigations have fully established the ancient doctrine that fevers, properly so called, are idiopathic and *general* diseases, involving more or less functional derangement of all the organs; while the same investigations have equally demonstrated the falsity of the theories invented for their explanation, without establishing any others more satisfactory in their stead. It is true, that some, like Dr. Stevens, place the primary link in the chain of morbid action, in the blood; others adopting the ideas so clearly and logically set forth by Dr. Southwood Smith, trace the first morbid impression to the nervous system; while the great majority content themselves with tracing, as carefully as possible, the causes or circumstances under which these diseases arise, their distinctive varieties, the changes that take place in the various organs and tissues during their progress, and the effects of treatment, leaving the question of their essential pathology entirely untouched. This absence of any established or generally received ideas of the pathology of fever, has given rise to systems of treatment the most various, and often contradictory.

Indeed, there is no subject in the whole range of practical medicine, concerning which our medical literature furnishes so perfect a specimen of confusion and anarchy of opinion, as in relation to the one now under consideration.

For abundant proof of this, we need not look beyond the able report on the "Blending of the Types on Fever," and the reports on epidemics, published in the last volume of the Transactions of the American Medical Association. It is no part of my present object, however, to pursue these general observations, or to collate the diversified opinions of the profession concerning this subject.

Having occupied, during the last three or four years, a geographical position, where almost every variety and type of fever is to be met with, from the simplest form of *periodical* endemic, to the graver forms of *continued* fever, as they occur among the poorer class of emigrants, I have been induced to study them, both in hospital and private practice, with much interest and care. The result of this study is the conviction that fever, though diverse in its causes, phenomena, and results, and exceedingly complex in its composition, is yet susceptible of *analysis*, and capable of a rational and consistent explanation. To accomplish this, however, we must commence our study with a full comprehension of the extent of the subject, and the elements it embraces; otherwise we shall almost inevitably be led to partial views and erroneous conclusions.

Some have sought in a study of the causes of these diseases, an explanation of their nature and varieties; others like Broussais, Louis, Chomel, Gherard and Jackson, have sought the same end by the most minute and patient examination of the post mortem appearances; while a still larger number have fixed their attention on the symptoms or direct phenomena presented during life. Some of the latter, like Bœrhaave have spent much time in collecting and comparing all the symptoms of fever, detailed by different writers, with a view of determining which of them were common and invariably present in all the cases and varieties of the disease, a task which resulted in finding no such symptom; the only *three* left by Bœrhaave himself, having been long since found frequently absent. Others, instead of searching directly for the symptoms that might be constantly present, in all the forms of fever, have, with much labor and perseverance, collated the symptoms in each variety, stated their relative or numerical degree of prevalence or constancy and thereby endeavored to point out the specific differences between the several varieties. The most recent and important attempt of this kind has been made by Dr. Austin Flint; the results of which are found in his valuable work entitled, "Clinical Reports on Continued Fever."

This work, though containing a large amount of interesting and valuable matter, instead of throwing any light on the essential pathology of the continued Types of Fever, or establishing any fixed and constant differences between the two leading varieties, (Typhoid and Typhus,) does in fact only demonstrate that certain leading symptoms are more frequently present in one variety than in the other. In other words, instead of finding specifically different symptoms, those belonging to each variety of continued fever, bear the mathematical relation to each other of *plus* and *minus*. Another class, adopting the rules of investigation laid down by Dr. Southwood Smith, have diligently endeavored, first to ascertain, not the particular symptoms, but the particular organs and tissues whose functions were invariably disturbed in all varieties of fever; and second, the precise order in which these disturbances take place.

Dr. Smith himself asserts very positively that "there never was a case of fever," in which the *nervous* and *sensorial* functions, the *circulating* function, and the functions of *secretion* and *excretion* "*were not more or less in a morbid state.*" This, however, is simply a more systematic mode of expressing the well-known fact, that fevers are accompanied by universal disturbance of function, though very variable in the kind and amount of derangement in different cases. Concerning the second endeavor, there is much less unanimity of sentiment. Some regard changes in the *blood* as the *first* and essential step in the development of fever, such as the deficiency of the saline constituents, alteration of the properties of the corpuscles, &c. Others vaguely refer the first step to some morbid condition of the *Vital* properties of the solids; while Dr. Martin Payne boldly claims that it consists essentially and invariably in an "*exaltation* of *irritability* and *mobility*;" properties which he represents as elementary and vital. Much the larger number of writers at the present day, however, like Dr. Southwood Smith, attribute the first link in the chain of morbid action, to some unnatural impression on the nervous system, and make this the antecedent, if not the immediate cause of the subsequent disorder of the circulating and secreting structures. This doctrine is fully set forth in the paragraph already quoted from the last edition of Dr. Wood's Practice.

And yet the same able writer, in the same paragraph, is compelled to acknowledge that there is something, even in its initial step, "which differs from other instances of *nervous* depression." It seems to me, that all these modes of investigation have failed to fully accomplish the object for which they were instituted; first, from the too restricted or partial system of investigation pursued; and second, from a disregard of that analytical and inductive method of study, which is so necessary to guard against vague and erroneous conclusions.

If it is true that disease consists in a deviation from the healthy or natural state, of some one or all of the structures and functions of the human system; and if it is equally true that fevers, properly so called, essentially involve more or less deviation from the natural condition, of all the functions of the body, it would follow as an almost necessary inference, that there must be some property or properties capable of being acted on, which are so common or universally identified with all the tissues, that their disturbance necessarily disturbs in some degree the functions of the whole.—Hence, if we would successfully unravel the complicated phenomena of fevers, and obtain for ourselves clear ideas of their true pathology, we must not only study, critically and impartially, the causes, the symptoms, and the condition of the solids and fluids during life, as exhibited at the bed side, and the post mortem appearances, but we must include with all these a close analytical study of that condition of the fluids and those properties of the solids, the disturbances of which are capable of inducing equal disturbance of all the functions of the body.

In the further prosecution of my present task, I shall not enter upon the long protracted discussion, concerning the general or local character of fevers; for however frequent may be the complications of fever with local inflammation, I think no impartial observer can long maintain his position at the bedside of the sick, or traverse daily the clinical wards of a Hospital, without recognising, not only a marked distinction between fevers and inflammations, but also, the *idiopathic* and *general* character of the former, just as clearly as he does the local and limited character of the latter.

When, a few years since, I first took up and read that excellent work of Dr. C. J. B. Williams, entitled "Principles of Medicine," I felt no less disappointment than regret, that in his interesting study of the *elements* of disease, he should wholly omit the subject now under consideration. It then occurred to me that the same elementary and analytical process applied to the study of fevers, would contribute much to a clearer and more definite knowledge of these forms of disease; an impression which has since been much strengthened by clinical observation and study.

For the sake of clearness and accuracy, I shall propound the following distinct questions, and discuss them in the order in which they are stated, viz:

1st. Are there any properties, conditions, or forces possessed by all the living organized matter of the human body, whether constituting a part of the solid tissues or floating in the fluids, capable of being acted on by morbific agents in such a way as to produce general functional disturbance of the whole system?

2nd. Are all the varied phenomena necessarily belonging to the different types of fever, when closely analyzed, susceptible of being satisfactorily explained, by a knowledge of the action of morbific agents, on the properties or forces alluded to in the preceding question?

That all living organized matter, whether vegetable or animal, is possessed of certain properties, which are inherent and essential, constituting its vitality, seems to be a proposition universally assented to.

And yet, concerning the nature, origin and number of those properties, there is great diversity of opinion, and much confusion in the modes of expression adopted by different authors.

Thus says Dr. G. B. Wood in his summary of general pathology, which introduces his valuable work on Practical Medicine; "All living parts are endowed with the property of excitability, or, in other words, are capable of being brought into and maintained in action by the stimulus of certain agents, either internal or external." In the same paragraph, however, he confounds this property of excitability with the function of ordinary sensation, as follows: "But under the influence of certain agents, this excitement may be increased or diminished so as to occasion *uneasiness* to the individual, and interfere with the due performance of the functions. Thus heat and cold, in the ordinary changes of the seasons, often produce *painful* impressions, the former with an augmented, the latter with a diminished action of the part affected." But the confusion does not stop here. In the following paragraph, taken from page 16, vol. i., he not only uses the words "nervous energy" as synonymous with "excitability," but represents both as existing in definite *quantity*, capable, like the blood, of being driven from one part, and accumulating in another. Thus he says: "When the healthy actions of a portion of the frame are reduced, the *nervous energy* and circulating fluid, being diminished in that part, necessarily *accumulate elsewhere;* and if the cause continues to operate, a morbid excess of action, in other words an irritation, in some other part or organ is the consequence. This results, in part, from a mere change in the balance of the circulation, throwing an undue portion of blood, which is itself a powerfully stimulating agent, upon a particular organ; but probably in part also from the operation of that physiological law by which the system, *possessing a certain amount of excitability*, experiences a temporary increase of this property in certain parts, when its exercise is restrained in others."

Drs. Kirk and Paget enumerate three distinct *Vital* properties, viz.: Formative Force, Contractility or Irritability, and Vis Nervosa, or nervous power. Dr. Williams and Carpenter, speak of Contractility or Irritability and Tonicity, as Vital properties, but limit their existence to muscular or contractile tissues. While the latter well known Physiologist, seems to claim but one general vital property, which he styles *Vital Force*, and labors to show that even it is strictly co-relative with ordinary physical and chemical forces. Thus he says: "It seems, then, to be a legitimate expression of the dynamical conditions, requisite for the production of the phenomena which we distinguish as *Vital*, to say that they are dependent, directly or indirectly, upon the *Physical force* pervading the Universe; which, acting through organized structures as their "Material Substratum," manifest themselves as Vital Force, one of the most characteristic operations of this being the production of new tissue, which in its turn may become the instrument of a similar metamorphosis. *And we have the same kind of evidence, that light and, heat acting on the organic germ become transformed into Vital Force*, which we possess of the conversion of Heat into Electricity by acting on a certain combination of metals, or of Electricity into Magnetism by being passed round a bar of iron, or of Heat or Electricity, into motion, when their self-repulsive action separates the particles from each other."*

With becoming deference for so learned an author, I must express the opinion, that in this sentence, and in all his comments on the subject embraced in it, he has not only failed to explain anything, but has remarkably intermixed and confounded things essentially different. That light and heat when brought to act in proper force, on a *living* organic germ, will give rise to actions

* See Carpenter's Principles of Human Physiology, page 143–4.

which are termed *Vital*, is universally admitted. But why are they termed *Vital?* Simply because they exhibit phenomena and results peculiar and different from anything that we see in inorganic matter. And why these peculiarities? Not certainly from any peculiar property or transformation of the light and heat; but plainly from a peculiar susceptibility or determinative quality possessed by the *organic germ* itself. A susceptibility or quality which must not only exist in the germ, as a necessary antecedent, but which can alone render the action of light and heat of any avail in producing vital phenomena. Thus you may pour light and heat on the unfecundated egg as long as you please and no vital actions will result. There must, therefore, be an anteceden susceptibility in the germ, on which the light and heat, and all other stimuli act; and this it is that constitutes the true Vital Force. Hence to say, with Carpenter, "that light and heat acting on the organic germ, become transformed into Vital Force," is only another mode of stating the absurd proposition, that organic or vital actions are the result of light and heat acting on some modification of themselves.

Dr. Martin Paine enumerates six vital properties, viz: "Irritability, Mobility, Vital Affinity, Vivification, Sensibility, and Nervous Power." A part of these, like Sensibility, are so plainly *functions* of a particular structure, instead of elementary vital properties that they need no comment.

Drs. Niel and Smith in their Hand Book of Physiology, state, "that two vital properties are described by Physiologists, to wit: Sensibility and Contractility." They very properly add, however that Iritability or Contractility may be considered the *only* vital property possessed by all living beings.

Dr. Brown used the term "Excitability," but in such a sense as to include both sensibility and irritability; while many English, French, and German Physiologists, like Drs. Cullen and Gregory, and Drs. Eberle and Wood of our own country, seem to regard this property as something derived from the brain and the nervous system

According to Dr. Samuel Jackson of Philadelphia, "the term Irritability was first introduced into medicine by Dr. Francis Glisson," who was followed by DeGorter, Winter and Haller; while

Stahl, Hoffmann and others attributed the same phenomena to a kind of intelligent agency personified as the Vis Anima, &c. Dr. Jackson himself claims two vital properties, viz: "Organic Force or Irritability, and Vital Affinity."* These allusions to different writers, clearly show two things: First, that the idea of the existence of some vital property or properties, existing in all organized matter, by which such matter, is rendered capable of being acted on by external agents, is very generally, if not universally entertained. Second, that there is no general agreement as to the number or nature of such properties, or the part they play in maintaining health or giving rise to the phenomena of disease.

A somewhat extended series of investigations have led me to regard *two* conditions as necessary and essential for the production of Vital phenomena. The first consists in a peculiar arrangement of the particles of matter, which, for want of a better term, I shall style Tonicity, or texture. The second consists in a peculiar property which I shall call Susceptibility. Let us view living matter in whatever aspect we may, whether in the simple vegetable germ, or the more complex animal tissue, a rigid analysis will invariably lead us to a definite texture or Tonicity, and a Susceptibility, as inherent, elementary and universal conditions, without the possession of which there can be no manifestation of life, no vital action. Of the essential nature of these two conditions we know nothing, inasmuch as they are ultimate and elementary; bearing the same relation to living organic matter, that the elementary physical forces termed caloric, attraction &c., do to inorganic matter.—That they do not consist of any mere modification of the ordinary physical forces, is evident from the fact that every germ or tissue or cell must have an antecedent possession of them, or the operation of ordinary physical forces, produces only decomposition instead of vital action. That they are not derived from any connection with the nervous system, is equally evident from the fact that they exist in seeds and germs where no nerves exist, and also from the fact that the nervous power or influence, like the physical forces, can only act on matter possessing an *antecedent susceptibility*.

Hence, I state, it is a fundamental physiological maxim, that all

---

* See Jackson's Principles of Medicine.

living matter possesses two inherent, elementary conditions, viz: a definite Tonicity, or arrangement of particles constituting its structure, and a Susceptibility, by which it is capable of receiving the impressions of external agents. Thus while the primary cell of the vegetable or animal germ, retains these conditions in their normal state, it only requires a proper arrangement of the external or physical agents, such as heat, light, moisture &c., to excite in it that action which causes it to assimilate other matter to itself, developing new cells and all the ordinary active phenomena of organic life.

But let either of these conditions be destroyed or essentially modified, as by the simple temporary exposure of the germ cell to too high a temperature, and no possible arrangement of external or physical agents will ever after succeed in eliciting from it one single *Vital* phenomenon. Physiologists have not always made clear distinctions between elementary vital properties, and primary functions.

Many, like Dr. Payne, speak of Contractility, Mobility, and Sensibility, as elementary vital properties; when they are really elementary functions of particular parts. Thus, matter arranged in the form of the primary muscular fibre and possessed of Susceptibility, will *contract* on the application of nervous energy, electricity, or other stimulants. Hence, Contractility or Contraction is the specific or primary function of the muscular fibre. Its necessary antecedents and coincidents are, a particular Tonicity or texture, a Susceptibility, and the impression of a stimulant or excitant. In the same sense, *Sensibility* is a specific and primary function of nerve matter. A definite structure consisting of nerve cells or fibres and a Susceptibility, renders the action of a suitable agent capable of inducing what we term Sensibility.

If the nerve cells or fibres constitute a part of the nervous system of animal life or of relation, the sensation will be recognized by the mind, and designated as pleasurable or painful, and perhaps in return an energy or agency, will be sent to certain muscular fibres exciting their elementary function of contraction; from which results definite and intelligent motions. If they constitute a part of the nerves of organic life, the mind will be entirely unconscious of any sensation; yet an impression, which might appropriately be styled an organic sensation, is transmitted to an appropriate centre from which emanates an influence to corresponding organic or involuntary muscular fibres. Hence *Sensibility* and *Transmissibility* are the Specific functions of nerve fibres, whether they belong to the system of ordinary sensation and voluntary motion, to that which is so intimately connected with respiration and circulation, or to that more obscure system which links the various organs together and brings the whole into close sympathetic relation. The same analytic examination will show the true position of Nutrition, Secretion, &c. Thus the presence of a nutritive fluid in contact with any tissue possessed of its natural tone or texture and Susceptibility, will excite that peculiar molecular action, which results in the deposite of new particles, and the removal of old ones. So, too, the presence of a fluid containing the proper ingredients in contact with secreting cells or tubules, induces such changes as result in the production of a new fluid termed a secretion. We thus see that nutrition and secretion, like sensibility, transmissbility, and contractility, are elementary functions of particular structure, while all are alike dependent on the antecedent, and universally inherent properties or conditions which have been styled Tonicity and Susceptibility.

The disciplined and reflecting mind will trace these properties and functions in all living matter, from the simplest form of animal existence, presenting only the two elementary conditions, and a single primary function—nutrition—to the more complex organs of the higher orders of the animal kingdom, in which several of the elementary functions may be combined to produce some of the more complex phenomena of life. Thus, a simple voluntary movement, requires at least two elementary functions, viz.: transmissibility in the nerve, and contractility in the muscular fibre.

Having now answered, as definitely as time and space will permit, the question whether there are any properties or conditions, possessed alike, by all the living organized matter of the human body, the next inquiry is, whether such properties or conditions, are capable of being acted on by morbific agents, in such a way as to produce general functional disturbance of the whole system? It is a fundamental and fatal error of the Physioiogical or Broussaian School of Medicine, that after announcing irritability or susceptibility to be an inherent property of all living matter, capable of being acted on by excitants or stimuli, they passed at once to the uncalled for conclusion that the action of all such agents is local; and consequently that all disease must be local also. On the contrary, if Susceptibility and Tonicity are universally inherent properties, capable of being modified in one part or tissue then certainly, by the extension of the same or similar causes, they would be capable of modification, at the same time, in all the tissues. If the first would be productive of local functional derangement, the last would equally produce *general* functional derangement. The fact, however, that both Tonicity and Susceptibility are capable of being modified by the action of certain agents, in all the organs and tissues of the body simultaneously, rests on no mere hypothetical reasoning; but is established by a great variety of observations. Among the most familiar of these observations, are the effect of those all-pervading physical agents or forces, termed caloric and electricity. So directly does the first of these agents affect the elementary vital properties of all organized matter, that if we expose the germinating seed or the fecundated egg, to a temperature only a few degrees above blood heat, no subsequent treatment will restore their susceptibility, or render them capable of exhibiting vital phenomena. Equally familiar is the fact that the tissues of the human system, when exposed to a high temperature, become soft or flacid—in other words, their *tonicity* becomes diminished, and their susceptibility becomes, in nearly the same ratio, increased; while a low temperature produces just the reverse effect on both. Even non-professional men habitually notice this change in their own physical systems, with the changes of the seasons.

Another mode, less familiar, but not less important, by which the vital properties become modified, consists in the alterations either in the quantity, quality or composition of the blood. The blood, perpetually circulating and constantly present in every tissue, and in contact with every cell and fibre of the body, can undergo no unnatural or morbific change without inducing an effect simultaneously, though not perhaps to the same extent, in all the tissues. When the circulating fluid exists in full quantity and is rich in Corpuscles and nutritive matter, the individual enjoys what is called "high health." The flesh is firm, the circulation and and muscular contractions are vigroous and well sustained, and the nervous and secreting structures are active, indicating general strength and vigor without undue susceptibility. But let the quantity of the blood be deficient, as from excessive hemorrhages, and the flesh soon looses its firmness, the muscles their strength and power of endurance, and the secretions diminish' while the susceptibility or irritability frequently becomes increased. But perhaps the most striking and direct proof of the power to alter the inherent property of Susceptibility, by altering the quality and composition of the blood, is afforded by the effects of injecting Saline and other solutions into the veins of patients laboring under Epidemic Cholera. When, under the influence of that rapid disease, the organic susceptibility has almost ceased, the pulse being no longer perceptible at the wrist, the skin being shrivelled and purple, the visceral secretions and production of animal heat being all suspended, the injection of a few ounces of some Saline solution into the veins, generally produces an effect so speedy and universal throughout the whole system as to surprise the observer. In a very few moments the pulse returns in the radial artery, the capillary circulation improves, the skin becomes less shrivelled, the eyes less sunken, the skin and tissues again become warm, and the kidneys and other secreting organs take on their natural functions. It is true that this improvement is too often only temporary; for with the general improvement of organic susceptibility, comes also a renewal of the gastric and intestinal irritations or determinations, with the usual copious discharges and consequent recollapse. This however does not lessen the value of the experiment, as proof that the inherent susceptibility of the tissues is capable of increase and diminution by altering the condition of the blood. The power to act directly on the all pervading property of living matter, which we have styled Susceptibility, is further illustrated by the very recent experiments of Dr. Brown Sequard on the effects of renewing the circulation through the vessels of limbs entirely severed from the body. That the effects in all these cases are produced by a direct action on the elementary and inherent property or properties of the tissues, and not, as some might suppose, on the organic nerves, is evident from two considerations. First, the substances used to inject the veins, in the collapsed stage of Cholera, were not such as usually produce any perceptible effect on the nervous system, and in Dr. Brown Sequard's experiments there was no possibility of making an impresssion on the nervous filaments belonging to the cerebro spinal system, because the limbs experimented on were not only cut off or severed entirely from the body, but were allowed in several instances to become quite cold and rigid. Second, there are some substances which, when taken into the system, very obviously possess the power to excite or exhilarate the nervous system, while at the same time, they diminish both Tonicity and Susceptibility; as indicated by a diminution of the capillary circulation, the secretions, the animal heat, the power of endurance, and the consciousness of organic suffering, while at the same time, the excitement of the brain and nerves produces in the individual a deceptive feeling of warmth, activity and strength. Perhaps the most familiar substance of this class, and one that displays its double influence on the human system most clearly, is alcohol. This substance, when diluted in the form of distilled spirits, and taken into the stomach, rapidly enters the blood unchanged, and in a few minutes perceptibly modifies almost every function of the system. It causes a feeling of exhilaration or excitement to pervade the brain, and thereby modifies the actions of the mind. At the same time, the respiration and the pulse become slightly quickened, but less full and forcible; the secretions diminish, as indicated by the dryness of the mouth and fauces; the proportion of Carbonic Acid in the expired air is diminished; the tone and steadiness of the muscular fibres become impaired; and the temperature of the system, as indicated by the thermometer, under the tongue, actually becomes lowered. If the impression made by Alcohol was on the nervous system alone, the excitement or exhilaration of that tissue should be accompanied by corresponding exaltation of the functions of all the other tissues; but numerous experiments demonstrate the reverse to be true.

Observing several years since, a wide and irreconcilable difference or discrepancy between the theoretical and popular idea that alcohol and alcoholic drinks, from their highly carbonaceous composition, were among the most efficient supporters of animal heat and respiration, and the actual physical effects of those substances on human health, strength and power of endurance, as unequivocably displayed in their daily and extended use throughout civilized society,—I commenced, in the winter of 1850, a series of experiments, designed positively to determine the effects of different classes of food and drinks on respiration and calorification.

These experiments have been continued up to the present time, and their results will be made manifest hereafter.

But the effects of alcoholic drinks so strikingly illustrate some of the positions taken in the annexed essay, that I cannot forbear detailing one or two experiments with the accompanying remarks.

*Experiment* 1. On the 18th of October, 1852, after remaining quiet two hours in a room, the temperature of which was kept steady at 70° Farenheit, at $8\frac{3}{4}$ o'clock, P.M., my temperature as indicated by a delicately graduated Thermometer, the bulb of which was inserted under the tongue, was $98\frac{1}{4}$°, F. the pulse 76 per minute, and respirations 17. I took at once three ounces of brandy mixed with water. *Thirty* minutes after the brandy was taken, I felt an unusual dryness in the mouth and fauces, a feeling of exhilaration in the head, with a slight general feeling of numbness throughout the whole system, and a sensation of increased heat especially in the stomach and face; the thermometer, applied in the same manner as before, indicated the temperature at 98° F., the pulse 84, and respiration 17. *Sixty* minutes after taking the brandy, all the feelings just mentioned were much increased; the sense of exhilaration in the head amounting to decided giddiness or feeling of intoxication; the temperature was $97\frac{3}{4}$° F., pulse 77, and respiration 17.—*One hundred and twenty* minutes after taking the Brandy, all the feelings before described had begun slightly to diminish; the temperature was $97\frac{1}{2}$° F., the pulse 75, and respirations 16.

(*Continued in next No.*)

